# Influence of Lipid Composition on Nonspecific Interactions of Serotonin with Model Membranes

**DOI:** 10.1021/acsptsci.5c00767

**Published:** 2026-01-29

**Authors:** Jamie Gudyka, Jasmin Ceja Vega, Jessica Said, Shakinah Silverberg, Amani Rabadi, Jacqueline Ceja, Wilber Perla, Christopher Poust, Elizabeth Andersen, Joseph Mitchell, Mackenna Agosti, Giovanna Mazzo, Sunghee Lee

**Affiliations:** Department of Chemistry and Biochemistry, Iona University, 715 North Avenue, New Rochelle, New York 10801, United States

**Keywords:** serotonin, model membrane, water permeability, differential scanning calorimetry, confocal Raman microspectroscopy, interfacial tensiometry

## Abstract

Serotonin is a monoamine neurotransmitter, which plays an important role in the development and functioning of the central nervous system. Recent biophysical studies reveal that nonspecific interactions between serotonin and lipid membranes significantly alter lipid bilayer properties, impacting synaptic function and plasticity. To better understand these critical interactions and their broader implications for neural function and pharmacology, we investigated the interactions of serotonin (at concentrations ranging from 1 to 40 mM) with model membranes prepared as droplet interface bilayers, liposomes, and supported bilayers. These membrane systems comprised single, binary, and ternary lipid mixtures, including pure DOPC, DOPC/DOPS (10:1 mol ratio), and DOPC/Sphingomyelin/Cholesterol (1:1:0.2 mol ratio). Our analysis employing various experimental techniques shows that the interaction of serotonin with lipid membranes of diverse compositions has overall nonspecific effects in (1) influencing the barrier properties of the lipid membrane, as demonstrated by increased water permeability compared to the control; (2) modifying the phase transition behavior, evidenced by decrease in the main phase transition temperature and reduction of the transition enthalpy; (3) perturbing the conformational ordering of lipid membranes, as indicated by the increase in specific Raman intensity ratio; and (4) reducing bilayer tension with increasing serotonin concentrations. Overall, membrane modifications increase with rising serotonin concentrations, plateauing at higher levels. Sensitivity to serotonin varies by lipid composition in the order: DOPC/DOPS ≈ DOPC/Sphingomyelin/Cholesterol > DOPC. Our experimental findings reveal that serotonin significantly alters membrane properties, particularly affecting neuronal membrane composition and lipid rafts, which are critical for membrane protein organization and signaling. These findings suggest that serotonergic drugs and pathological fluctuations in serotonin may influence signaling not only through classical receptor-mediated pathways, but also by altering the lipid–protein landscape of the membrane, with potential implications for drug efficacy, off-target effects, and the development of therapies that target membrane composition in serotonin-related disorders.

## Graphical abstract

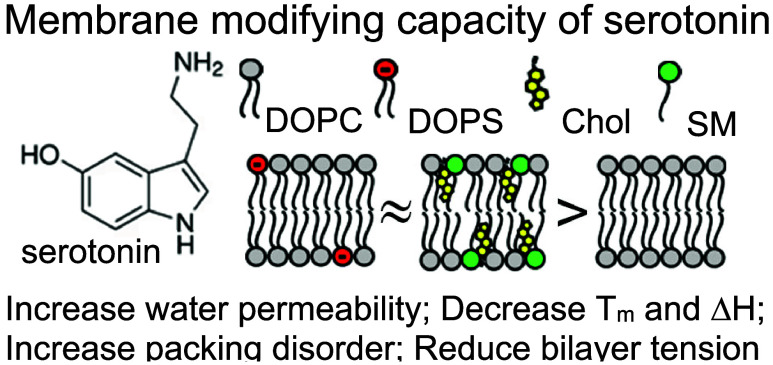

Serotonin is a monoamine neurotransmitter derived from tryptophan that plays an important role in the development and functioning of the central nervous system. It regulates mood, cognition, and diverse physiological functions; disruptions in serotonin synaptic function have been correlated with various mental disorders such as depression, anxiety disorders, and other behavioral issues.^1,2^ A traditional understanding of the serotonin synapses in the brain has been mainly focused on specific proteins that coordinate synthesis, storage, release, receptor interactions, and degradation of the neurotransmitter.^3^ However, recent research has shown that synaptic membranes are highly adaptable in their composition and physical and mechanical properties, and that the actual function of membrane-bound proteins specific to serotonin synapses is actively regulated by their lipid environment, posing a question to the largely protein-centric view and suggesting consideration of a lipid bilayer-mediated mechanism.^2^

The pivotal role of lipids in cellular processes is increasingly evident, with mounting research demonstrating that membranes are not merely passive structures but active participants in regulating protein functions, facilitating signaling cascades, and orchestrating protein sorting in various essential physiological processes.^4–6^ In particular, the organization of lipids into distinct domains within the plasma membrane can significantly impact the localization, conformation, and activity of membrane-associated proteins.^6^ Recent advances in biophysical research have unveiled the significant impact of nonspecific interactions between neurotransmitters and lipid membranes, demonstrating that these interactions can induce substantial alterations in the physical properties of lipid bilayers. These membrane alterations, in turn, can profoundly influence the structure and dynamics of membrane-embedded proteins, modulating neurotransmitter diffusion and ultimately reshaping the overall synaptic neurotransmission process.^2,7^ Specifically, recent computational and experimental studies have provided evidence suggesting that, in addition to direct binding of serotonin to its specific receptors, serotonin interacts significantly with lipid bilayers and modulates membrane properties.^8–11^ This nonspecific interaction has been reported to take place at concentrations relevant to physiological conditions, with serotonin adhering to the hydrophilic–hydrophobic interface located between the lipid headgroups and the hydrocarbon tails of lipid bilayers.^12^ Additionally, studies have demonstrated serotonin’s ability to intercalate into the lipid membrane, positioning itself within the glycerol region between adjacent lipid molecules, to change the membrane properties of the lipid bilayer.^13^ Through a combination of atomistic molecular dynamics (MD) simulations and experimental studies, it has been observed that serotonin binds to the anionic phosphate group in phosphatidylcholine (PC) by interaction through serotonin’s cationic primary amine. This interaction positions its aromatic ring in alignment with the hydrocarbon tails, thus affording protection against lipid peroxidation.^14^ Moreover, serotonergic drugs, including psychedelics, have been reported to directly interact with and alter the physical properties of lipid bilayers, independent of their well-known actions at serotonin receptors.^15,16^ A recent report also demonstrates that serotonin can allosterically modulate the activity of a noncognate G-protein-coupled receptor (Y4R) by altering the physical properties of the surrounding lipid membrane, thereby reducing the receptor’s ligand affinity without directly binding to the receptor itself.^17^ Serotonin has also been reported to enhance the association and fusion of lipid vesicles, indicating its role in a lipid bilayer-mediated mechanism during the neurotransmission process.^18^ AFM studies have revealed that serotonin’s impact on membrane mechanical properties is dependent on lipid composition.^10^ Previous studies have shown that lipid rafts are critical for regulating serotonin transporters, as their localization within these microdomains influences transporter activity and signaling.^19^ Additionally, the integrity of lipid rafts is essential for the proper functioning of the serotonin type 3 (5-HT_3_) receptor; disruption of these domains reduces receptor stability and impairs signaling, underscoring the importance of lipid rafts in neurotransmitter receptor function.^20^

Investigating how serotonin interacts with bilayers of varying lipid composition provides valuable insight into membrane-mediated mechanisms of signaling and underscores the importance of considering both nonspecific membrane effects and receptor-specific interactions in rational pharmaceutical design targeting serotonin receptor function and dynamics.^21^ Such a multifaceted approach is essential for advancing our knowledge of neurotransmitter-membrane interactions, synaptic plasticity, and the development of more precise therapeutic agents. Accordingly, this study explores how serotonin alters membrane properties and examines how different lipid compositions influence this process. Because plasma membranes contain a highly diverse mixture of lipids,^22,23^ model membrane systems are utilized to achieve systematic control over membrane composition. This approach enables direct investigation of how lipid composition influences nonspecific interactions with serotonin, without the confounding presence of proteins.^24^ The lipid compositions of model membranes used in this study are shown in Table 1 with their chemical structures in Figure 1. We selected 1,2-dioleoyl-*sn*-glycero-3-phosphocholine (DOPC) to form a single-component, neutral zwitterionic lipid membrane as its fluid phase at physiological temperatures, and well-characterized physical properties make it widely used for studying membrane structure, dynamics, and interactions.^25,26^ A binary mixture of DOPC and 1,2-dioleoyl-*sn*-glycero-3-phospho-l-serine (sodium salt) (DOPS) was selected to represent the overall lipid composition of the synaptic membrane, capturing the balance between zwitterionic PC and the anionic phosphatidylserine characteristic of neuronal membranes.^27–29^ Synaptic vesicles are predominantly composed of PC (∼40 mol %) with a smaller fraction of PS (∼10 mol %).^28^ In addition, the extracellular leaflet of a synaptic membrane is often modeled using a ternary mixture of DOPC, sphingomyelin (SM), and cholesterol (Chol) to mimic the characteristic lipid raft domains.^30,31^ Membrane rafts are dynamic, cholesterol- and sphingolipid-enriched domains that form the liquid-ordered phase (l_o_) dispersed within the liquid-disordered phase (l_d_) of the cell membrane and have been implicated in diverse cellular processes, including cell signaling, membrane protein organization, and other vital functions.^32–36^ The selection of the DOPC/SM/Chol 1:1:0.2 molar ratio was driven by relevance to membrane raft studies. At this composition, the phase diagram demonstrates coexistence of liquid-disordered (l_d_) and liquid-ordered (l_o_) phases, which is essential for modeling raft-like domain formation and behavior.^37,38^ This ratio also enables the investigation of thermotropic properties relevant to membrane rafts. Importantly, higher cholesterol concentrations abolished the main phase transition in differential scanning calorimetry (DSC) measurements, thus precluding analysis of phase transition behavior. Serotonin, also known as 5-hydroxytryptamine (5-HT), has a chemical structure comprising a tryptamine ring with a hydroxylated ethylamine side chain. The experiments were conducted by using serotonin hydrochloride.

**Table 1:** Lipid Component of Model Membranes Used in This Study

lipid composition (mol)	model membranes
DOPC	common zwitterionic lipid membrane in mammalian cell membranes
DOPC/DOPS (10:1)	anionic lipid membrane associated with synaptic membranes
DOPC/SM/Chol (1:1:0.2)	extracellular leaflet of a synaptic membrane (membrane rafts)

**Figure 1:**
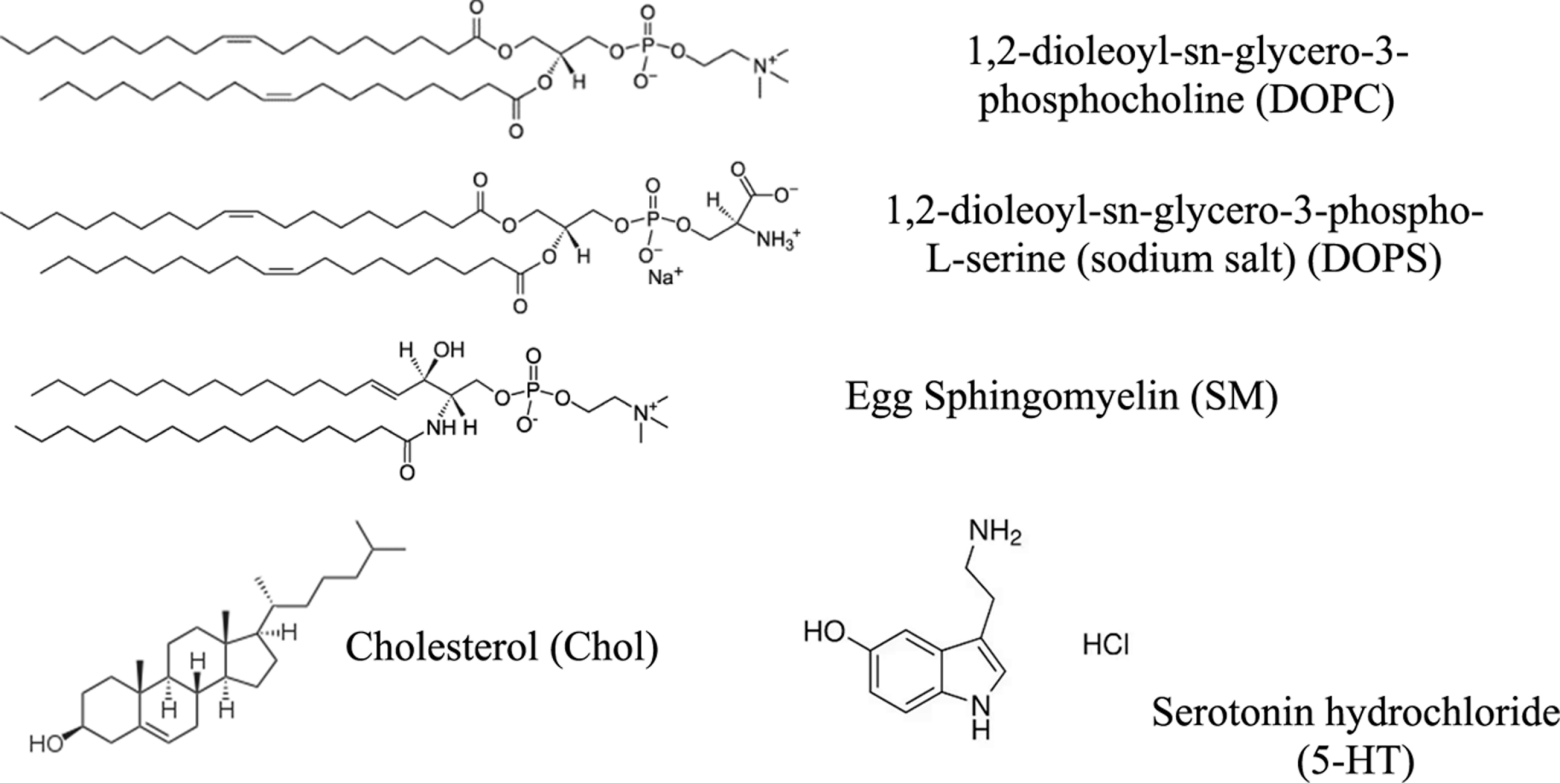
Chemical structure of molecules used.

To investigate the nature and extent of nonspecific interactions between serotonin and model lipid membranes of diverse compositions, this study employs a combination of complementary experimental techniques, including measurements of transbilayer water permeability to detect alterations in barrier properties, DSC to probe thermotropic behavior, confocal Raman microspectroscopy to assess conformational order, and bilayer tension to evaluate the interfacial properties over a serotonin concentration range of 1 to 40 mM. The average serotonin concentration in all types of synaptic vesicles is reported to be about 270 mM,^39^ emphasizing the high local availability of serotonin in the synaptic environment and motivating the investigation of serotonin’s concentration-dependent effects on membrane properties. Specifically, we aim to examine how serotonin concentration influences passive water permeability, determine how lipid composition modulates this effect, and relate these permeability changes to structural properties revealed by the different techniques.

## Results and Discussion

### Serotonin Increases Water Permeability of Model Lipid Membranes

We employed the droplet interface bilayer (DIB) platform as our biomimetic model for cellular membranes to probe whether serotonin affects passive permeation of water through selectively permeable bilayer assemblies. Gaining insight into membrane permeation is critically important, as the movement of small molecules across bilayer membranes profoundly influences cellular physiology and homeostasis.^40,41^ The DIB is formed when aqueous microdroplets bounded by lipid monolayers create a bilayered region upon contact.^42,43^ This interdroplet contact zone closely resembles the double-leaflet lipid bilayers and provides a versatile platform for biomimetic modeling of cellular membranes, with unique opportunities to study intriguing chemistry at the nanoscale level of lipid bilayer.^44,45^ In our earlier studies, we established a DIB-based method to assess the barrier properties to passive water permeation.^46,47^ By employing water as a molecular probe, we demonstrated that the water permeation rate across a bilayer is greatly influenced by both the physical condition and compositional differences of cell membranes.^48–50^ Furthermore, our previous studies reveal that a wide range of biologically active molecules are capable of sensitively modulating the structure of lipid membranes, thereby affecting their transbilayer water permeabilities and other biophysical properties.^51–56^ Thus, investigating water permeability can greatly enhance our understanding of the innate barrier properties exhibited by different lipid membranes and highlight the intricate relationship between composition, structure, and properties in biological membranes with diverse compositions.^44^

Figure 2 illustrates the typical image sequences for a DIB-based osmotic water permeability experiment. The detailed methods for the water permeability determination are provided in the Experimental Section and Supporting Information. Figure 2 shows a pair of aqueous droplets: the leftmost droplet contains serotonin at a specified concentration in 0.1 M NaCl, while the rightmost droplet contains pure water (Figure 2A). Both droplets are in immiscible oil (squalene, SqE) containing lipid molecules and undergo water transport across the DIB in the presence of the osmotic gradient, resulting in a measurable diameter change of the leftmost droplet (swelling) and the rightmost droplet (shrinking), as shown in Figure 2B.

**Figure 2:**
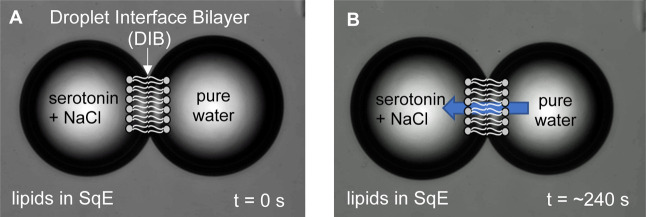
A DIB mimics the cell membranes via self-assembly of amphiphilic lipids: it consists of a double leaflet of lipids organized into a lipid bilayer structure. Image sequences (A,B) for a typical DIB-based water permeability experiment show osmotic water transport across DIB blue arrow in (B) indicates the direction of water transport. Each droplet is about 100 μm in diameter at its initial size.

Table 2 shows the osmotic water permeability coefficients (*P*_f_) of model membranes of different lipid compositions at 30 °C in the serotonin concentration range of 1–40 mM. Water permeability values are reported as the mean of at least 50 individual measurements, with error bars representing the standard deviation (mean ± SD).

**Table 2:** Osmotic Water Permeability at 30 °C of Model Membranes of Different Lipid Compositions as a Function of Serotonin Concentrations^a^ ^,^ ^b^

	water permeability coefficient, *P*_f_ (μm/s), at 30 °C avg ± SD		
serotonin concentrations (mM)	DOPC	DOPC/DOPS (10:1)	DOPC/SM/Chol (1:1:0.2)
0	73 ± 2	62 ± 2	67 ± 2
1	75 ± 2	63 ± 2	68 ± 3
5	76 ± 3	65 ± 3	74 ± 3
10	78 ± 2	71 ± 3	76 ± 4
20	79 ± 4	73 ± 4	78 ± 3
40	83 ± 3	74 ± 3	80 ± 4

a Note that before the addition of serotonin, the water permeabilities for model membranes differ depending on their lipid compositions: 73, 62, and 67 μm/s for DOPC, DOPC/DOPS (10:1), and DOPC/SM/Chol (1:1:0.2), respectively, as shown in Table 2. Studies have shown that DOPS bilayers are thicker and have a smaller cross-sectional area compared to DOPC bilayers,^57^ with DOPS also having a lower molar volume than DOPC.^58^ DOPC and DOPS are known to mix ideally in multilamellar vesicle dispersions, and the incorporation of DOPS into DOPC bilayers results in a decrease in the area per molecule.^59^ Since water permeability is closely related to lipid bilayer properties such as area per lipid and bilayer thickness,^60^ it is anticipated that model membranes composed of DOPC doped with DOPS would exhibit lower water permeability compared to pure DOPC membranes, as shown in our study. The water permeability of a ternary mixture of DOPC/SM/Chol (67 μm/s) is lower than that of a single-component DOPC membrane (73 μm/s). Our observations of reduced water permeability in the ternary system align with previous findings on similar lipid mixtures. For instance, studies on DOPC/SM/Chol (1:1:1) ternary systems containing microdomains have reported that these membranes are less permeable to water than would be predicted based on their lysis tensions.^61^

b Water permeability data represent an average of individual permeability runs (*n* ≥ 50) and standard deviation as error bars (mean ± standard deviation).

Figure 3 shows the relative osmotic water permeability coefficients, expressed as *P*_f_/*P*_f_^°^, where *P*_f_^°^ is the osmotic water permeability in the absence of serotonin at 30 °C; all values are normalized to their respective controls with control values set to 1. Statistical analyses were conducted using a two-way ANOVA with Tukey’s post hoc tests to (i) assess the effect of concentration within each membrane model and (ii) evaluate differences among membrane models at identical serotonin concentrations. Significance levels are indicated in Figure 3.

**Figure 3:**
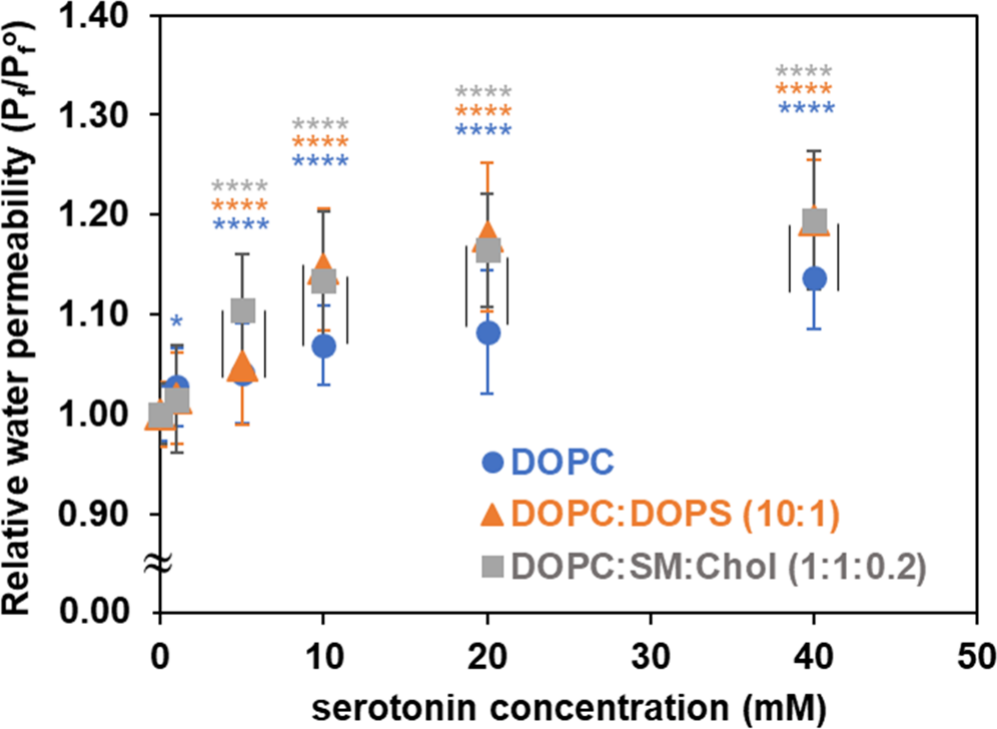
Relative osmotic water permeability coefficients (*P*_f_/*P*_f_^°^) are shown for model membranes formed from DOPC (blue circle), DOPC/DOPS (10:1) (orange triangle), and DOPC/SM/Chol (1:1:0.2) (gray square), at 30 °C with varying serotonin concentrations. All values are normalized to their respective control means. Error bars for the control represent the relative standard deviation of the raw control measurements prior to normalization. Data were analyzed using two-way ANOVA, followed by Tukey’s post hoc tests to compare water permeability (*P*_f_) between serotonin concentrations and their respective controls within each membrane model (colored stars), and among the three membrane models at identical serotonin concentrations (black vertical lines). Only significant differences (*p* < 0.05) are shown: colored stars, *p < 0.05, ****p < 0.0001; black vertical lines for *p* < 0.0001.

As shown in Table 2 and Figure 3, the addition of serotonin to various lipid membranes reveals a general increase in water permeability in a concentration-dependent manner: the higher the concentration of serotonin, the greater the water permeability. The increase in water permeability becomes apparent even at lower serotonin levels, ranging from 1 mM to less than 10 mM. At and above a 10 mM serotonin concentration, the increase in relative water permeability (*P*_f_/*P*_f_^°^) shown in Figure 3 is more prominent compared to controls and shows clear lipid composition dependency: a greater increase in water permeability for membranes composed of DOPC/DOPS (orange triangles) and DOPC/SM/Chol (gray squares), than for DOPC (blue circles). In all cases, the rise in water permeability seems to level off as the serotonin concentration increases.

Our findings demonstrate that serotonin increases water permeability across three different model membranes, albeit to varying degrees, underscoring serotonin’s capacity to modulate the biophysical properties of lipid membranes in a composition-dependent manner. Overall, the permeability of water through bilayers is linked to diverse structural and physical characteristics of individual lipids and the resulting bilayer configuration. These characteristics include bilayer thickness, molecular packing density, polyunsaturation, and the overall fluidity of the membrane.^48,60,62,63^ Typically, the fluidity or rigidity of bilayers aligns with the packing density of lipids, suggesting that water permeability is indeed affected by lipid packing within the bilayer region.^50,64^ For example, a recent study, based on MD simulations of both POPC and POPS membranes, shows that serotonin in concentrations ranging from ∼0.5 to ∼5 mM significantly increases water penetration into the hydrophobic part of the lipid bilayer, a finding further corroborated by fluorescence studies.^18^ In addition, MD simulations have demonstrated a strong binding of serotonin to DMPC or DOPC membranes. This binding has been reported to result in decreased lipid chain ordering, attributed to the attraction between the primary amine of serotonin and the lipid phosphate group.^8^ The effect of serotonin on the order parameter has been measured by ^2^H NMR, revealing a significant perturbation in the acyl chain region, with a greater extent of decrease observed for POPS compared to POPC.^10^ Neutron reflection studies show that serotonin nonspecifically adsorbs to zwitterionic (POPC) and anionic membranes (POPC/POPG), and it intercalates into the bilayer with increased accumulation of serotonin to anionic bilayers due to electrostatic interaction, considering that serotonin is a cation at physiological pH.^12^ Additionally, the interaction of serotonin with raft-like mixed model membranes has been reported to lead to several changes, including increased membrane disorder, expansion of disordered domain, membrane softening, and domain composition alteration. Specifically, the lipid structure and packing of a ternary membrane mixture of POPC/SM/cholesterol (4:4:2) show a restructuring of the domains in the presence of 9 mol % serotonin, studied by both NMR and MD simulations.^65^ Serotonin is reported to decrease the lipid chain order in the disordered phase while increasing the lipid chain order in the ordered phase, thereby inducing hydrophobic mismatch between ordered and disordered phases that lead to the formation of larger size domains.^9,65^ Studies using atomic force microscopy (AFM) have shown a notable reduction in indentation force when serotonin (5 mM) is introduced to a membrane composed of a 1:1:1 molar ratio mixture of POPC/POPG/cholesterol.^13^ Additionally, ^2^H NMR measurements have revealed an increase in lipid chain disorder and a consequent decrease in the hydrophobic thickness of the membrane under these conditions.^13^ These findings collectively demonstrate serotonin’s capacity to alter key physical properties of complex lipid membranes and are consistent with the observed increases in water permeability.

### Serotonin Modifies Thermotropic Properties of Model Lipid Membranes

Using DSC, we demonstrate that the thermotropic properties of membranes are influenced by the serotonin concentration and lipid composition. Representative endothermic thermograms showing the effects of varying serotonin levels on different model membranes in MLVs are presented in Figure 4. Tables S1–S3 in the Supporting Information provide the thermodynamic data corresponding to Figure 4. The introduction of increased concentrations of serotonin modified the phase transition behavior of pure DOPC MLVs (*T*_m_ = −16.68 °C, Δ*H* = 8.34 kcal/mol for the transition from the lamellar gel phase L_β_ to the lamellar liquid-crystalline state L_α_ of DOPC, which is consistent with literature data^66^). Figure 4A and Table S1 show that higher concentrations of serotonin alter the main phase transition behavior of the DOPC, broadening the peak, shifting it toward lower *T*_m_, and resulting in an overall reduction of the transition enthalpy. These effects are more pronounced at relatively higher serotonin concentrations. When serotonin is introduced in the bilayer at 100 to 1 mol ratio of DOPC to serotonin, *T*_m_ slightly decreases by 0.14 °C compared to pure DOPC, with no significant reduction in Δ*H* (∼1% reduction) and no change in peak width. These values change only gradually with increasing molar ratios of serotonin, and at 4 to 1 mol ratio, the highest concentrations tested in this study, the main phase transition shifts to a lower temperature by 1.1 °C (from −16.68 to −17.78 °C) accompanied by a reduction in enthalpy (from 8.34 to 5.31 kcal/mol) and a broadening of the peak width (from 1.5 to 2.51 °C). Previous studies have reported that the addition of serotonin induces a reduction in the *T*_m_ of DMPC membranes, accompanied by a generally broadened transition profile;^8,67^ notably, these findings—obtained using saturated acyl chain PC—are qualitatively consistent with our observations.

**Figure 4:**
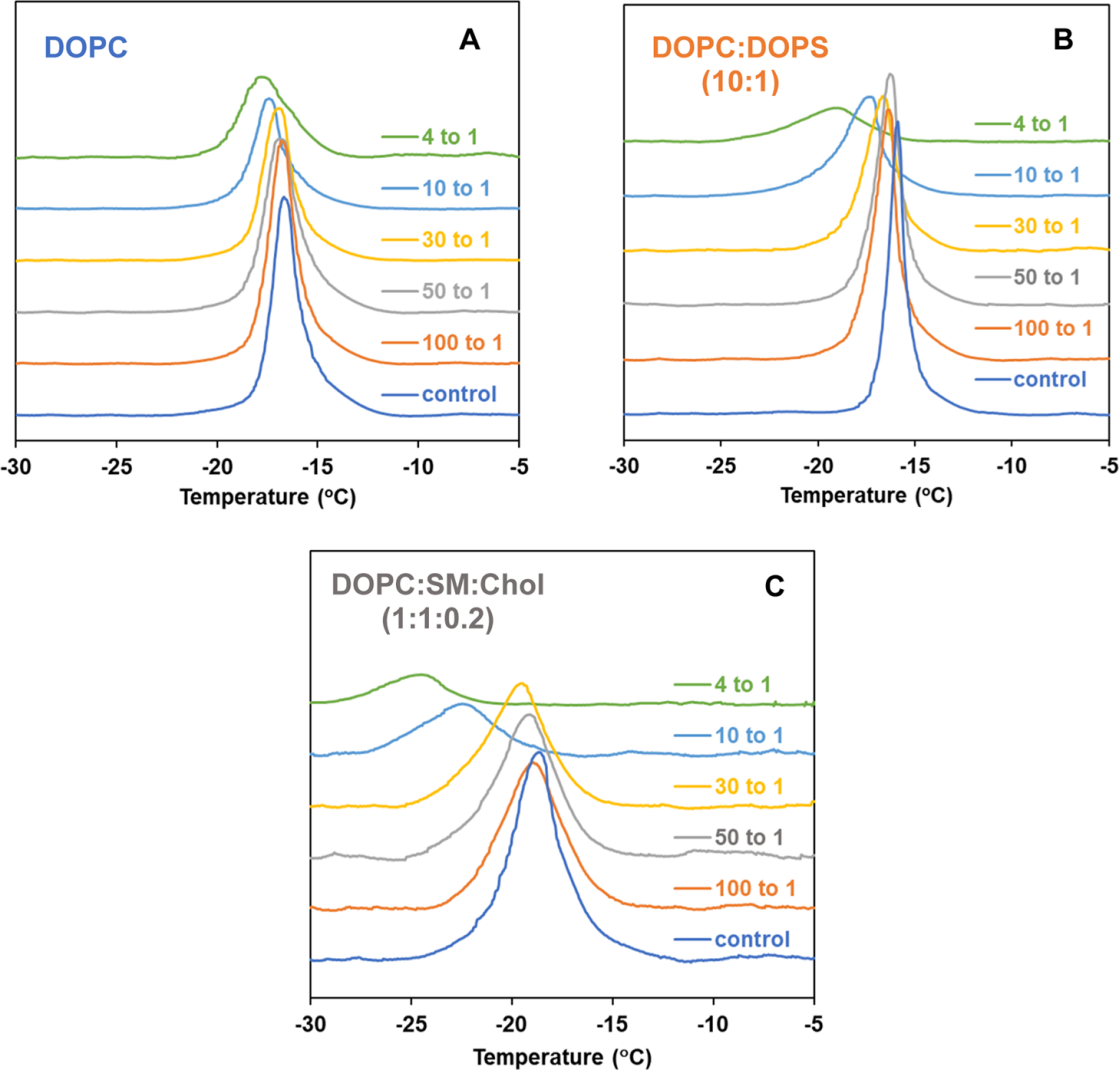
Endothermic thermograms of MLVs of (A) DOPC, (B) DOPC/DOPS (10:1), and (C) DOPC/SM/Chol (1:1:0.2) MLVs with varying concentrations of serotonin. The corresponding thermodynamic data are shown in Tables S1–S3 in the Supporting Information.

In Figure 4B and Table S2, the endothermic thermograms of DOPC/DOPS (10:1 molar ratio) MLVs are depicted along with their modifications in the presence of different concentrations of serotonin. Incorporating 10 mol % DOPS into DOPC resulted in a shift toward a higher *T*_m_ temperature at *T*_m_ = −15.88 °C (compared to −16.68 °C for pure DOPC), a greater enthalpy with Δ*H* = 9.10 kcal/mol (compared to 8.34 kcal/mol for pure DOPC), and a narrow bandwidth of 0.95 °C (compared to 1.50 °C for pure DOPC), aligning well with the reported condensation effect of DOPS that leads to a tighter packing of lipid bilayer mixtures.^59^ Similar qualitative trends to DOPC MLVs are observed, with increasing concentrations of serotonin leading to a shift to lower temperatures, broadening the peak, and reducing the transition enthalpy; notably, the extent of these changes is greater than that observed with DOPC alone at high concentrations of serotonin. At a 4:1 mol ratio, the main transition shifts to a lower temperature by 3.10 °C (from −15.88 to −18.98 °C), accompanied by a reduction in its enthalpy from 9.10 kcal/mol (control) to 3.30 kcal/mol and a broadening of the peak width from 0.95 °C (control) to 3.74 °C.

The endothermic thermograms of ternary DOPC/SM/Chol (1:1:0.2 molar ratio) MLVs in the absence of serotonin (Figure 4C, blue trace) exhibit a single but broad transition (fwhm = 2.73 °C), indicative of the heterogeneous nature of the mixture (Figure 4C bottom blue trace). This transition, representing the shift from the lamellar gel phase (L_β_) to the lamellar liquid-crystalline state (L_α_) of DOPC, is observed at *T*_m_ = −18.67 °C with a significantly suppressed enthalpy of 2.67 kcal/mol (Table S3). Notably, no SM liquid crystalline phase transitions were detected (scan up to 65 °C) in this ternary mixture, which is consistent with previous reports.^68^ The main phase transition of the ternary mixture shifts to a lower temperature (a decrease of about 2.21 °C from −18.67 to −22.36 °C) at a 10 to 1 molar ratio of lipid mixtures to serotonin, with a significant reduction in transition enthalpy from 2.67 to 1.17 kcal/mol. At a 4:1 molar ratio, a further shift of the peak is observed (to −24.54 °C) with a reduction in enthalpy of transition (0.45 kcal/mol). A broadening of the peak width is observed with increasing concentrations of serotonin, ranging from 2.73 °C (control) to 5.50 °C. The observed broadening of the endothermic transition in all three membrane models can be interpreted in terms of the localization of serotonin within the bilayer. Specifically, occupancy of the C1–C10 methylene region places a guest molecule near the cooperative lipid segments, where it perturbs local packing and increases heterogeneity in the headgroup region.^69,70^ This perturbation reduces the cooperativity of the phase transition, resulting in a broader, less distinct endothermic peak that spans a wider temperature range.

For a relative comparison, Figure 5A shows the changes in main phase transition temperature (*T*_m_–*T*_m_^°^), where *T*_m_^°^ and *T*_m_ are the main phase transition temperatures in the absence and presence of given serotonin concentrations, respectively, for different model membranes, as a function of mole fraction of serotonin. Similarly, Figure 5B shows the relative enthalpy of the transition (shown as the ratio Δ*H*/Δ*H*°) as a function of the mole fraction of serotonin, where Δ*H*° is the transition enthalpy in the absence of serotonin. As seen in Figure 5A,B, serotonin interacted with model membranes of various lipid compositions, leading to alterations in the bilayer’s thermotropic properties and affecting the packing arrangement of lipid acyl chains in a concentration- and composition-dependent manner. Overall, qualitatively similar trends in the change of the DOPC thermogram are observed for model membranes composed of various lipid compositions, that is, a decrease in the transition temperature (Figure 5A) and a reduction in the transition enthalpy (Figure 5B). However, the extent of the decrease in *T*_m_ and Δ*H* with increasing content of serotonin depends on the membrane composition, with the greatest modification observed in membranes consisting of DOPC/SM/Chol, followed by DOPC/DOPS, and the least changes seen with DOPC.

**Figure 5:**
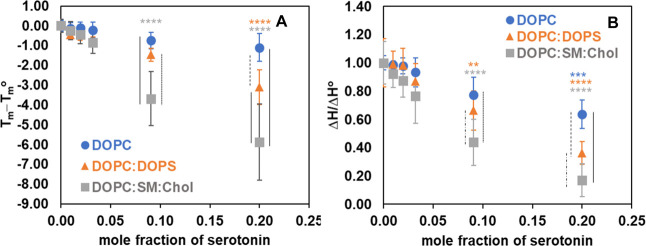
Effect of serotonin on the thermotropic parameters (A) change in main phase transition temperature and (B) relative enthalpy of different model membranes. All values in B are normalized to their respective control means. Error bars for the control represent the relative standard deviation of the raw control measurements prior to normalization. Statistical comparisons of Δ*T*_m_ and Δ*H*/Δ*H*° were performed using two-way ANOVA, followed by Tukey’s post hoc tests to assess differences between serotonin concentrations and their respective controls within each membrane model (colored stars) and across the three membrane models at equivalent serotonin concentrations (black vertical lines). Only significant results (*p* < 0.05) are displayed: colored stars, **p < 0.01, ***p < 0.001, ****p < 0.0001; black vertical lines, solid for *p* < 0.0001, dotted for *p* < 0.001, dashed for *p* < 0.01, and dash-dot for *p* < 0.05.

Our findings regarding the effect of serotonin on the phase behavior of DOPC, DOPC/DOPS, and DOPC/SM/Chol membranes are qualitatively consistent with the results observed in water permeability (shown in Figure 3). This highlights the membrane-modifying effect induced by serotonin, which is dependent on the lipid composition. In the next section, we further investigate the effect of serotonin on the conformational order of lipid bilayers of varying compositions by using confocal Raman microspectroscopy.

### Serotonin Modifies the Acyl Chain Order of Model Lipid Membranes

In this section, we used the specific Raman peaks and their intensity ratios to evaluate the impact of serotonin on the conformational order of lipid membranes. The Supporting Information provides representative Raman spectra (650–3450 cm^–1^) of supported lipid bilayers with various lipid compositions as well as pure serotonin film, together with the corresponding peak assignments in Table S4 (Figure S1). Figure S2 shows the room temperature spectra of bilayers prepared by varying the amount of serotonin added. Figure 6A–C shows Raman spectra in the C–H stretching frequency region (2800 to 3000 cm^–1^) for three supported model membranes, in the absence and in the presence of serotonin at a 10 to 1 molar ratio of lipids to serotonin. All Raman spectra are normalized to the intensity at ∼2850 cm^–1^ for comparison. The C–H stretching region has been extensively studied in lipid membrane spectra due to its strong Raman scattering signal and high sensitivity to the order of acyl chains and the packing of lipids.^71^ The peaks at around 2850 cm^–1^ and 2890 cm^–1^ correspond to the symmetric (C–H_sym_) and antisymmetric (C–H_asym_) stretching modes of methylene groups, respectively. The peak at approximately 2930 cm^–1^ is assigned to the symmetric stretching mode for the acyl chain methyl (C–H_term_). It is widely recognized that these three peaks in the C–H stretching region, as well as their intensity ratios, are valuable indicators for determining the structural properties of membranes.^72–75^ Therefore, the spectral variations in this region, along with the intensity ratios (*I*) of [C–H_term_/C–H_asym_] and [C–H_term_/C–H_sym_], were utilized to detect conformational changes that resulted in modifications of the acyl chain order/disorder and lateral packing density in the three different model membranes upon interaction with serotonin molecules. As shown in Figure 6A–C, upon addition of serotonin (10 to 1 molar ratio of lipids to serotonin), the change in the Raman spectral features in the C–H stretching frequency region is apparent. Compared to a control (absence of serotonin, blue trace), the peak at ∼2890 cm^–1^ decreases in intensity (filled green arrow) and 2930 cm^–1^ increases in intensity (open green arrow) with the addition of serotonin (orange trace): the greater extent of such changes for DOPC/DOPS and DOPC/SM/Chol compared to that for DOPC.

**Figure 6:**
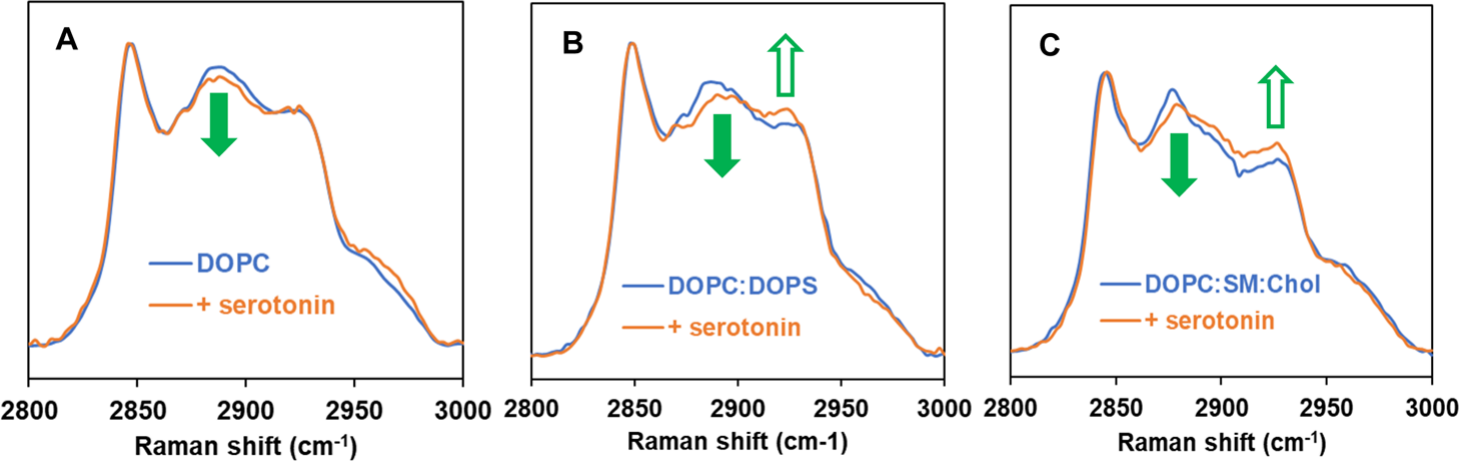
Representative Raman spectra of supported bilayers of different model lipid membranes at room temperature, (A) DOPC, (B) DOPC/DOPS (10:1), and (C) DOPC/SM/Chol (1:1:0.2) in the absence (blue) and presence (orange) of serotonin at a 10 to 1 molar ratio of lipids to serotonin. Green arrows highlight the change in intensity of the peak at ∼2890 cm^–1^ and ∼2930 cm^–1^.

Figure 7 illustrates the relative Raman intensity ratios, *I*/*I*_o_ (2930/2890), which report on the lateral packing density and intermolecular ordering of lipid acyl chains.^71^ *I*_o_ is 2930/2890 ratio in the absence of serotonin. The corresponding Raman intensity values are provided in the Supporting Information (Table S5). We also analyzed the I2930/I2850 ratio (Supporting Information), which displays an increasing trend similar to that of *I* 2930/*I* 2890, consistent with enhanced acyl-chain disorder and motional freedom in the bilayer. Note that there is a small contribution of serotonin-originated peak in the C–H stretching region (Figure S1). At high concentrations of serotonin-containing bilayers (e.g., 10 to 1 and 4 to 1), there is an increasing intensity of the peak at ∼1540 cm^–1^, which is attributed to the indole ring stretching vibration of this molecule (Figure S1 and S2).^76^ Therefore, the relative Raman intensity ratios shown in Figure 7 are the resultant ratios after subtraction of the serotonin-originated peaks from the spectra at these concentrations. As shown in Figure 7, higher concentrations of serotonin in membranes of DOPC/DOPS and DOPC/SM/Chol lead to a general increase in the intensity ratio of [C–H_term_ (2930)/C–H_asym_ (2890)] peaks, while such an increase is less pronounced for membranes consisting of DOPC. As the ratio increases with higher serotonin content, it becomes evident that greater serotonin association within the lipid bilayer affects the coupling between molecules in the acyl chain region and influences intermolecular interactions in the lipid membranes. This progressive reduction in packing order leads to a disordering effect on the lipid membranes. Consequently, the observed intensity changes are attributed to the sensitivity of the lipid bilayer to increasing gauche content. Higher values of the peak ratio [C–H_term_ (2930)/C–H_asym_ (2890)] indicate a decrease in both intramolecular (gauche/trans) and intermolecular (chain packing) interactions. These ratios reflect changes in acyl chain conformation, signifying increased rotations, twists, and bends that ultimately lead to packing disorder.^73^

**Figure 7:**
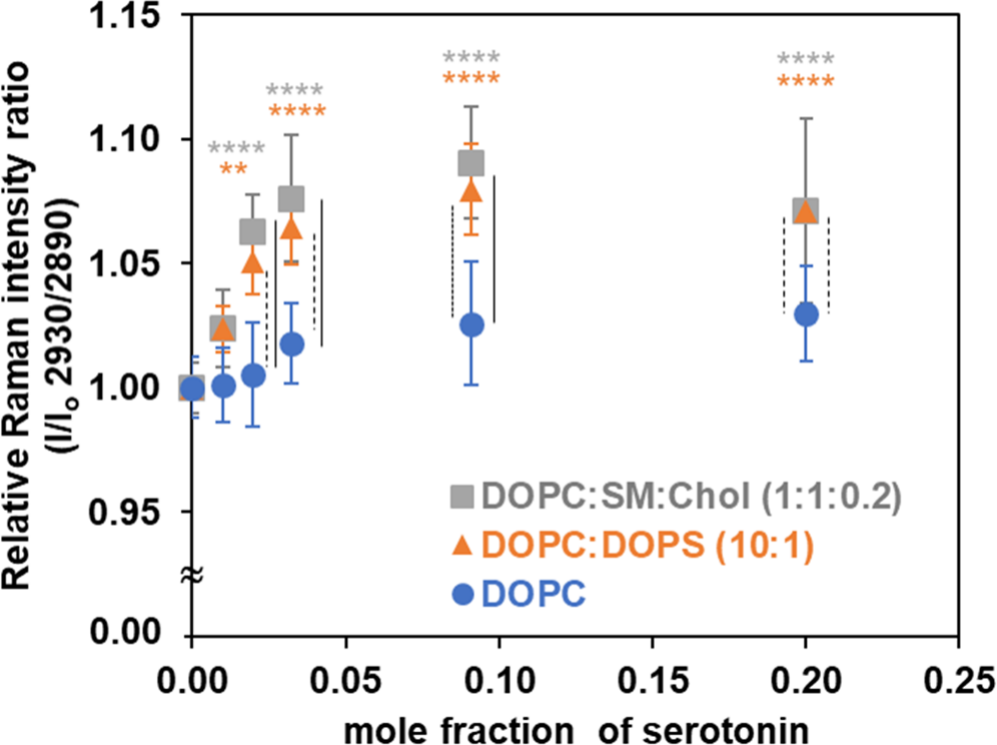
Relative Raman intensity ratios [*I*/*I*_o_ = *I* C–H_term_ (2930)/*I* C–H_asym_ (2890)] at room temperature are shown as a function of serotonin content, normalized to the mean value of the corresponding control. Each point represents the mean ± SD of three independently prepared samples, with three regions scanned per sample and averaged. The data were analyzed by two-way ANOVA with Tukey’s post hoc tests to compare Raman intensity ratios across serotonin concentrations and their controls within each membrane model (colored stars) and among the three membrane models at the same serotonin concentration (black vertical lines). Only significant differences (*p* < 0.05) are indicated: colored stars, **p < 0.01, ****p < 0.0001; black vertical lines, solid for *p* < 0.0001, dotted for *p* < 0.001, dashed for *p* < 0.01.

Overall, our Raman spectral analysis indicates increased acyl chain disorder, likely due to reduced lipid packing. This effect is qualitatively consistent with our earlier observations of increased water permeability and decreased phase transition temperature and enthalpy. We note that Raman spectroscopy provides a bulk measurement, averaging signals from all domains within the sample,^15^ which inhibits resolution of domain-specific changes, particularly in ternary membranes. Nevertheless, the data demonstrate that serotonin alters the structural properties of lipid membranes in a manner that is dependent on lipid composition.

### Serotonin Reduces Bilayer Tension of the DIB Interface

A fundamental parameter for understanding how bioactive molecules interact with lipid bilayers is bilayer tension, which reflects the surface energy across the membrane. Bilayer tension is closely tied to membrane rigidity and stability^77^ and plays a significant role in essential cellular processes such as membrane fusion and the activity of membrane-associated proteins.^78^ Many bioactive compounds, particularly those with amphiphilic character, interact with membranes in ways that manifest as measurable changes in bilayer tension, concomitant to the adsorption of such molecules at the interface. To quantify these effects, we combined interfacial tension measurements with interdroplet contact angle (θ) analysis in DIB systems.^45,79^ The monolayer interfacial tension (γ_m_), obtained using a pendant drop tensiometer, together with the contact angle formed between adherent droplets, allows direct calculation of bilayer tension (γ_b_) according to eq 1. This approach provides a robust means to assess the interfacial adsorption behavior of bioactive molecules.

1 $$\gamma_{b}={2\gamma }_{m}\cos\theta$$

Table 3 shows the effects of varying concentrations of serotonin HCl on monolayer tension, contact angle, and bilayer tension of membranes composed of DOPC and DOPC/SM/Chol (1:1:0.2 mol ratio).

**Table 3:** Interfacial Activities of Serotonin HCl at the Water/DOPC and Water/DOPC/SM/Chol(1:1:0.2)"DSC0.2"-Squalene Interface at 25 °C^a^

	monolayer tension, γ_m_ (mN/m)		contact angle, θ (degrees)		bilayer tension, γ_b_ = 2γ_m_ cos θ (mN/m)	
serotonin (mM)	DOPC	DSC0.2	DOPC	DSC0.2	DOPC	DSC0.2
0	1.125 ± 0.009	1.185 ± 0.027	31.20 ± 0.24	23.96 ± 0.34	1.92 ± 0.01	2.17 ± 0.04
1	1.082 ± 0.017	1.042 ± 0.022	29.80 ± 0.26	24.16 ± 0.34	1.88 ± 0.03	1.90 ± 0.04
5	0.982 ± 0.024	0.990 ± 0.025	30.89 ± 0.33	24.61 ± 0.20	1.69 ± 0.04	1.80 ± 0.04
10	0.931 ± 0.015	0.900 ± 0.009	33.90 ± 0.28	27.13 ± 0.38	1.55 ± 0.02	1.60 ± 0.01
20	0.864 ± 0.026	0.627 ± 0.018	40.35 ± 0.25	29.71 ± 0.33	1.32 ± 0.03	1.09 ± 0.03
40	0.664 ± 0.019	0.428 ± 0.029	48.71 ± 0.22	35.95 ± 0.73	0.88 ± 0.02	0.69 ± 0.04

a γ_m_ data represent average and standard deviation (SD) for *n* = ≥6 trials; θ data represent average and standard deviation (SD) for *n* = ≥10 trials.

The interfacial tension of the DOPC monolayer (γ_m_) decreased from 1.125 to 0.664 mN/m with increased serotonin concentrations from 0 to 40 mM. At the same concentrations, the contact angle between two aqueous droplets increased from 31.20° to 48.71° (Figure S3 provides images of DIB pairs at various concentrations of serotonin HCl in 0.1 M NaCl). Table 3 also shows that an increase in serotonin concentration in the aqueous phase engenders a reduction in bilayer tension, from 1.92 mN/m in the absence of serotonin to 0.88 mN/m in the presence of 40 mM serotonin. Also shown in Table 3 are the interfacial parameters for ternary component membranes (DOPC/SM/Chol at 1:1:0.2 mol ratio) in the presence of serotonin, which can be compared to the single component DOPC. An increase of serotonin concentration in the aqueous phase enhances the adsorption of serotonin at the bilayer interface consisting of DOPC/SM/Chol, as shown by a reduction in bilayer tension, from 2.17 mN/m in the absence of serotonin to 0.69 mN/m in the presence of 40 mM serotonin. For comparison, Figure 8 shows the relative bilayer tension, expressed by γ_b_/γ_b_°, where γ_b_ and γ_b_^°^ represent bilayer tension in the presence and absence of serotonin, respectively. Overall, qualitatively similar effects are seen upon inclusion of serotonin for both DOPC single component bilayers and a ternary component (DOPC/SM/Chol) bilayers, but with a greater reduction of bilayer tension for the latter, especially at high concentrations, at 20 and 40 mM.

**Figure 8:**
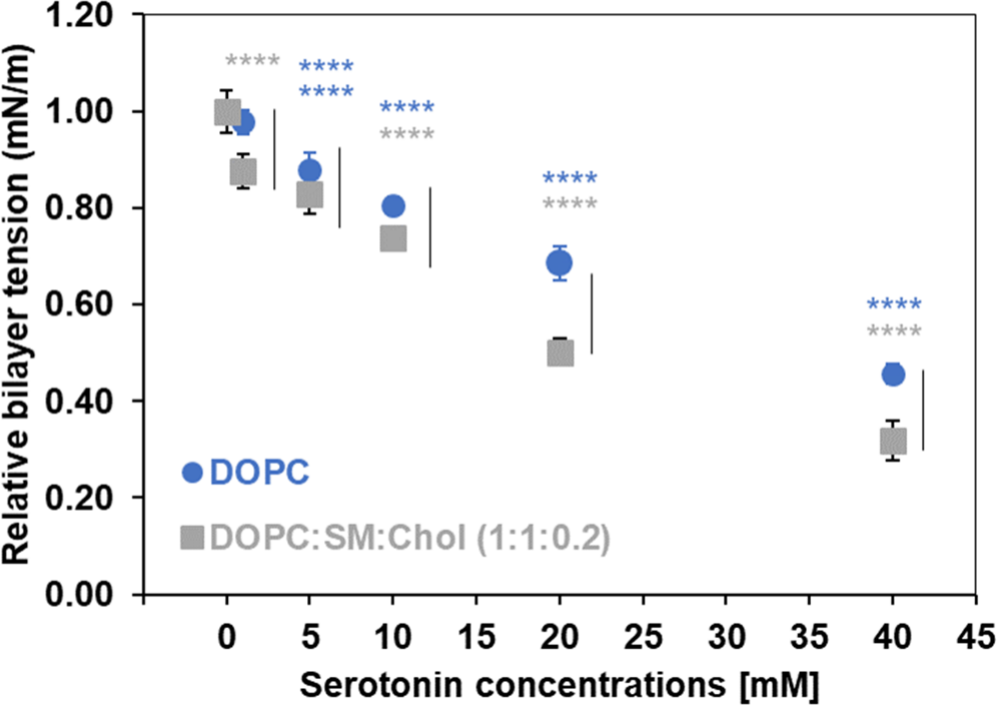
Relative bilayer tension upon exposure of increasing serotonin concentrations to DOPC (blue circle) and DOPC/SM/Chol (gray square) bilayers. Bilayer tension was analyzed by two-way ANOVA with Tukey’s post hoc tests to compare serotonin concentrations with their controls within each membrane model (colored stars) and between the two membrane models at the same serotonin concentration (black vertical lines). Only significant differences (*p* < 0.05) are shown: colored stars and black vertical lines, ****p < 0.0001.

Our data showing a relatively greater reduction in bilayer tension in the presence of serotonin concentrations for ternary component membranes is consistent with our observation of a greater increase in water permeability as well as greater reduction in *T*_m_ and Δ*H* by DSC spectroscopic studies and more disorder in the acyl chain packing parameters, as seen in the Raman spectroscopic studies.

## Conclusion

Our findings from complementary experimental techniques show that the interaction of serotonin with lipid membranes of diverse compositions has the following overall nonspecific effects: (1) influencing the barrier properties of the lipid membrane, as demonstrated by increased water permeability compared to the control; (2) modifying the phase transition behavior, evidenced by decrease in the main phase transition temperature, broadening of peak width, and reduction of the transition enthalpy; (3) perturbing the conformational ordering of lipid membranes, as indicated by the decrease in acyl chain ordering, leading to a packing disordering effect; and (4) decreasing bilayer tension induced by enhanced adsorption at the bilayer interface with increasing serotonin concentrations. The 1–40 mM serotonin range was selected to represent a biophysically relevant fraction of the high intravesicular levels (on the order of hundreds of millimolar)^39,80^ and to approximate the transiently elevated local concentrations^13^ experienced at serotonergic membranes during storage, release, and reuptake, rather than to mimic systemic clinical concentrations. Overall, the extent of modifications in the lipid membranes scales with the concentrations of serotonin, exhibiting somewhat leveling effects at high concentrations. The role of different lipid compositions is clearly demonstrated by the following order of sensitivity: DOPC/DOPS ≈ DOPC/SM/Chol > DOPC. Statistical analyses confirmed the observed trends, indicating that the biophysical changes reported here result from significant and interacting effects of membrane composition and concentration.

Changes in the properties of lipid membranes can influence the structure and conformational dynamics of proteins embedded within them. Such perturbation of embedded proteins can result in significant changes in their biological functions, including in homeostasis, protein activity, and the propagation of cellular signaling processes.^6,81–84^ For instance, modifications in domain composition or membrane fluidity can modulate the function of ion channels, receptors, and enzymes by affecting their conformational dynamics or assembly within the membrane.^6^ Furthermore, increased membrane permeability and altered domain structure may impact membrane fusion processes—essential for vesicular trafficking, neurotransmitter release, and other critical cellular events—with recent evidence indicating that serotonin can further modulate these fusion events by altering lipid bilayer properties, underscoring the critical role of the lipid environment in regulating fusion dynamics.^18^

Understanding how biologically active molecules interact with diverse lipid membranes and the resulting consequences for protein function is essential for elucidating the mechanisms of action and targeting of neurotransmitters and other modulators and may inspire innovative biomedical applications.^5,81^ Our results demonstrate that serotonin significantly modulates the physical properties of lipid bilayers of varying compositions, including ternary mixtures that mimic membrane rafts. Because such lipid domains are enriched in G protein-coupled receptors (GPCRs) and other key signaling proteins, serotonin-induced changes in these domains are expected to influence receptor conformation, ligand affinity, and signaling efficacy, with direct implications for synaptic transmission and drug action.^2,13,20^ For example, serotonin concentrations in patients are routinely manipulated by widely used drugs such as SSRIs, and dysregulated serotonin signaling is implicated in major depression, anxiety, migraine, and other neuropsychiatric and systemic disorders. By showing that serotonin can directly and differentially remodel membrane physical properties in a lipid composition-dependent manner, including in raft-like domains that organize GPCRs, transporters, and ion channels, these findings identify the membrane itself as a pharmacologically sensitive component of the serotonergic system.

The results further reveal that serotonin modulates membrane properties in a lipid-composition-dependent manner, even in the absence of receptors, supporting the emerging view that lipid bilayers are active participants in neurotransmission. In a pharmacological context, this suggests that serotonin and serotonergic drugs may act through dual mechanisms: classical receptor-mediated signaling and indirect modulation of receptor and transporter function via changes in the surrounding lipid matrix, particularly within raft-like microdomains. Such membrane-mediated effects may help explain concentration-dependent differences in efficacy and side effect profiles of serotonergic interventions and underscore the therapeutic potential of targeting membrane environments, alongside receptors themselves, to modulate serotonergic signaling in the nervous system. This membrane-centered perspective suggests modulation of lipid domains as a complementary strategy to traditional receptor- or transporter-targeted therapies in serotonin-related disorders. Future work will extend these studies to more complex, neuron-mimetic lipid mixtures to assess how the mechanisms identified here operate in compositions that more closely approximate native neuronal membranes.

## Experimental Section

### Materials and Sample Preparations

Lipids used in this study were obtained from Avanti Polar Lipids, Inc. (Alabaster, AL). 1,2-Dioleoyl-*sn*-glycero-3-phosphocholine (DOPC), 1,2-dioleoyl-*sn*-glycero-3-phospho-l-serine (sodium salt) (DOPS), and Egg Sphingomyelin (SM) were each provided as a solution in chloroform and used as received. According to the manufacturer, the acyl chain compositions of SM were 86% 16:0, 6% 18:0, 3% 22:0, 3% 24:1, and 2% unidentified species. Molecular structures of the lipids are shown in Figure 1. Squalene (2,6,10,15,19,23-hexamethyl-2,6,10,14,18,22-tetracosahexaene; C_30_H_50_; “SqE”) was used as the immiscible organic phase in the formation of DIB to determine water permeability coefficient, and all other chemicals, including cholesterol (Chol) and serotonin hydrochloride (5-HT), were of the highest purity available and were purchased from Sigma-Aldrich and used without additional purification. For sample preparations, a chloroform solution of lipidic components was evaporated under inert gas to produce a dried thin film of lipid (or lipid mixture). This was followed by overnight vacuum drying to ensure complete removal of any residual solvent. All lipid and lipid mixture samples were stored at −20 °C until use and freshly prepared immediately before use in experiments. Pure SqE was stored in the temperature range of 2 °C–8 °C. For water permeability experiments, an aliquot of SqE is added to the dried lipid film to achieve a total lipid concentration of 5 mg/mL. SqE is used as immiscible phase since its molecules are excluded from the DIB bilayer due to their large molecular size, to form an essentially solvent-free DIB. For sample preparations used in DSC, a suspension of multilamellar vesicles (MLVs) was prepared as follows: the required amount of serotonin (stock solution in methanol) was codissolved with the lipid mixture in chloroform. The solvent was then completely evaporated to produce a dried film of serotonin/lipid with a defined molar ratio of lipid to serotonin. The dried serotonin/lipid film described above is then rehydrated with deionized water (DI water) to achieve a total lipid concentration of approximately 16 mg/mL. The mixture is vortexed for about 5 min, followed by bath sonication for approximately 30 min, to produce MLVs. To prepare samples for confocal Raman microspectroscopy, the suspension of MLVs described above is subjected to seven freeze–thaw cycles using liquid nitrogen. All reported serotonin concentrations are expressed as molar ratios of lipid to serotonin, corresponding to the composition of the samples used for liposome preparation. All sample containers were wrapped in aluminum foil to prevent exposure to light. Aqueous solutions containing osmolytes (NaCl at nominally 0.1 M) were prepared from deionized water (18.2 MΩ·cm) purified in a Millipore water purification system (Direct Q-3). The osmolality (in mOsm/kg) of all aqueous solutions used was measured by a vapor pressure osmometer (VAPRO model 5600) immediately after fresh preparation of each solution, as well as prior to use.

### Water Permeability Using DIB

Water permeability measurements were conducted using a model membrane assembled by the DIB method. The detailed experimental setup and procedure have been described elsewhere.^49^

Briefly, a pair of osmotically unbalanced aqueous droplets, one containing pure water and the other an aqueous droplet of 0.1 M NaCl (and serotonin at a given concentration), each with a diameter of approximately 100 μm, are dispensed from a micropipet into a SqE solvent containing lipids or lipid mixtures. When two osmotically unbalanced microdroplets, each coated with lipid, were brought together to form a bilayer, osmotic water transport began immediately through the bilayer (contact zone), leading to a noticeable change in the droplet diameter. The osmotic gradient is responsible for driving the transport of water through the droplet bilayer. The flux of electrolytes is expected to be insignificant compared to that of water, as ion permeation is typically approximately 8 orders of magnitude slower than water permeation.^85^ Changes in the droplet size resulting from this water transport were measured. The detailed method for determining the water permeability coefficient is provided in the Supporting Information. All water permeability experiments were conducted at 30 °C using a custom-built, temperature-controlled microchamber. The chamber was maintained at the desired temperature with an external circulating water bath. Custom-built image analysis software was employed to postprocess the recorded videos and images, enabling precise measurements of droplet dimensions and contact areas.

### Thermal Properties Using DSC

Thermal phase transition analysis was conducted using a TA Q2000 Differential Scanning Calorimeter and analyzed with TA Universal Analysis software to study the main phase transition behavior of the samples. The main phase transition temperature, denoted as *T*_m_, corresponds to the temperature at the peak of the endothermic transition. The phase transition enthalpy was determined by integrating the area under the heat capacity curve. Additionally, baseline subtraction was performed by using OriginPro 9.7 software. A portion (approximately 15 μL) of the MLV suspension, prepared as outlined in the sample preparation section, was tightly sealed and subjected to a heating and cooling process at rates of 5 °C/min, ranging from −40 to −5 °C. This procedure was conducted under high-purity nitrogen with a flow rate of 50 mL/min. Reported values are averages ± the standard deviation from three sets of independently prepared samples. Each sample underwent three cycles to assess hysteresis, and reproducible results were obtained in all cases for consecutive heating and cooling cycles.

### Structural Properties Using Confocal Raman Microspectroscopy

Raman microspectroscopic investigations were carried out using an inverted confocal Raman microscope setup (XploRA INV, Horiba), which included a Raman spectrometer directly connected to an inverted microscope (Nikon Eclipse Ti-U). The Raman setup includes an internal laser kit operating at 532 nm (air-cooled solid-state laser) and a thermoelectrically cooled CCD detector. A 40× microscope objective (N.A. 0.60) was employed to focus a 532 nm laser beam and collect Raman-scattered light, which was subsequently dispersed by using a grating with 1200 lines per millimeter. The glass coverslips (#1.5) used as substrates for depositing lipid bilayer films were cleaned by rinsing with ethanol and drying with N_2_ before being used. Following this, a sample (10 to 20 μL) of a lipid or lipid mixture suspension, prepared immediately after the freeze–thaw process outlined in the sample preparation section, was applied to the cleaned coverslip surface. The aqueous solvent was evaporated in a sealed, homemade chamber. All Raman spectra were collected at ambient temperature. Typically, a data set consisting of 3 scans from different locations on the sample (across three separate samples) was averaged with 20 accumulations.

### Interfacial Properties Using Monolayer Tension and Contact Angle Measurement

Interfacial tension at the oil–water boundary was measured using a ramé-hart Advanced Goniometer/Tensiometer (model 590) equipped with DROPImage analysis software. These experiments investigated the surface adsorption behavior of DOPC and DOPC/SM/Chol mixtures at the squalene–aqueous interface. Different concentrations of serotonin HCl were added to the aqueous phase (0.1 M NaCl) to determine their impact on the interfacial properties. Typically, a 1 μL pendant droplet of the aqueous solution was suspended in about 1 mL of the lipid-containing oil phase. For contact angle (θ) measurements, two iso-osmotic droplets were manipulated into close proximity with micropipettes and then brought into contact. Supporting Information (Figure S3) provides the detailed methodology for contact angle measurement and images of DIB pairs (DOPC in squalene) at various concentrations of serotonin HCl in 0.1 M NaCl.

### Data Treatment and Statistical Analysis

Water permeability measurements were obtained from multiple individual runs (*n* ≥ 50) and are reported as mean ± standard deviation (SD). Recorded videos and images were analyzed by using custom-designed software to extract droplet dimensions and contact areas. DSC and Raman spectroscopic data represent the average of three separately prepared samples and are expressed as the mean ± SD. DSC thermograms were analyzed with TA Universal Analysis software to determine the main phase transition parameters, with baseline correction performed in OriginPro 9.7. Raman spectra were acquired and processed (background and baseline corrections) using LabSpec 6 Spectroscopy Suite Software (Horiba). Interfacial tension data were collected and analyzed using DROPImage Advanced software (ramé-hart). Statistical analyses were conducted using two-way ANOVA with a lipid membrane model (three types) and serotonin concentration (six levels, including control) as fixed factors. Water permeability, Raman intensity ratios, and phase transition parameters (*T*_m_ and Δ*H*) were analyzed as dependent variables, with *n* = 50 for water permeability and three independent replicates per condition for spectroscopic and calorimetric analyses. Tukey’s post hoc tests were applied for multiple comparisons.

## Supplementary Material



## Notes

The method of the determination of water permeability coefficient; DSC thermotropic data; Raman spectroscopic data and the Raman intensity ratios; and contact angle measurement and images of DIB pairs (PDF)

## Abbreviations

DOPC: 1,2-dioleoyl-*sn*-glycero-3-phosphocholine
DOPS: 1,2-dioleoyl-*sn*-glycero-3-phospho-l-serine (sodium salt)
SM: egg sphingomyelin
Chol: cholesterol
5-HT: serotonin hydrochloride
DIB: droplet interface bilayer
DSC: differential scanning calorimetry
SQE: squalene
MLV: multilamellar vesicle
