# Supplementary material for: Influence of Lipid Composition on Nonspecific Interactions of Serotonin with Model Membranes
Source: ACS Pharmacol Transl Sci. 2026 Jan 29;9(2):447–59. doi: 10.1021/acsptsci.5c00767 (PMC12910497; doi:10.1021/acsptsci.5c00767)
Supplement: Supplementary file 1 [file pt5c00767_si_001.pdf]

Supporting Information

Influence of lipid composition on nonspecific interactions of serotonin with model membranes

*Jamie Gudyka, Jasmin Ceja Vega, Jessica Said, Shakinah Silverberg, Amani Rabadi, Jacqueline Ceja, Wilber Perla, Christopher Poust, Elizabeth Andersen, Joseph Mitchell, Mackenna Agosti, Giovanna Mazzo, and Sunghee Lee**

Department of Chemistry and Biochemistry, Iona University, 715 North Avenue, New Rochelle, New York 10801, USA

*To whom correspondence should be addressed. Tel: 914-633-2638. E-mail: SLee@iona.edu

Text: Water permeability determination

Table S1. Temperature (T_m) and enthalpy (multilamellar liposome ΔH), for main phase transition of DOPC MLVs at varying concentrations of serotonin.

Table S2. Temperature (T_m) and enthalpy (multilamellar liposome ΔH), for main phase transition of DOPC:DOPS (10:1) MLVs at varying concentrations of serotonin.

Table S3. Temperature (T_m) and enthalpy (multilamellar liposome ΔH), for main phase transition of DOPC:SM:Chol (1:1:0.2) MLVs at varying concentrations of serotonin.

Table S4. Peak assignments of DOPC Raman spectrum.

Table S5. The corresponding Raman intensity ratios (I) of [$C-H_{term}$ (2930)/$C-H_{asym}$ (2890)] of membranes of different compositions at room temperature as a function of serotonin content.

Table S6. The corresponding Raman intensity ratios (I) of [$C-H_{term}$ (2930)/$C-H_{sym}$ (2850)] of membranes of different compositions at room temperature as a function of serotonin content.

Figure S1. A representative Raman spectra (normalized at ~2850 cm^{-1}) of pure DOPC, DOPC:DOPS (10:1), DOPC:SM:Chol (1:1:0.2), and pure serotonin film.

Figure S2. A representative Raman spectra of the (A) DOPC, (B) DOPC:DOPS (10:1), and (C) DOPC:SM:Chol (1:1:0.2) in the absence (blue) and presence of varying amount of serotonin.

Figure S3. The relative Raman intensity ratios (I/I_o) of [$C-H_{term}$ (2930)/$C-H_{sym}$ (2850)] of membranes at room temperature as a function of serotonin content.

Figure S4. Contact angle measurement and images of contact angle of a DIB pair (DOPC in squalene) in the presence of varying amount of serotonin.

Water permeability determination

The water permeability measurement was performed using the model membrane formed by the droplet interface bilayer (DIB) method. A DIB is formed when aqueous microdroplets bounded by lipid monolayers create a region that has a structure essentially the same as the double-leaflet lipid bilayer of cell membranes. When two osmotically unbalanced microdroplets were made to adhere at a bilayer, the osmotic gradient drives water transport through the droplet bilayer, resulting in a visible change in droplet diameter. Any electrolyte flux is expected to be negligible compared to that of water, as ion permeation is typically almost eight orders of magnitude slower than that of water. The corresponding changes in droplet volume over time (dV/dt) is measured optically by microscopic observation; and the behavior of the system follows the expression of equation (1) based on Fick's Law:

$$\frac{dV(t)}{dt} = -P_f A(t) \nu_w \Delta C(t) \qquad (1)$$

where A is the geometric bilayer area, ν_w is the molar volume of water (18 mL/mol), **Δ**C(t) is the osmolality gradient between two droplets, and P_f is the bilayer permeability coefficient of water. The volume change with time (dV/dt) is related to the bilayer permeability coefficient of water, P_f, as expressed in the Equation (1). When the bilayer contact area is constant, the time evolution of the swelling droplet can be obtained from the following equation derived from the integration of eqn. 1, with the following simplifying assumption: since one of the droplets (the shrinking droplet) contains no osmotic agent, its concentration does not change with time:[1, 2]

$$\left(\frac{V}{V_o}\right)^2 = \left(\frac{2P_f A v_w C_o}{V_o}\right) t + 1 \qquad (2)$$

Using the measured values for: initial size of the osmotic (swelling) droplet; bilayer contact area (A); and initial osmolarity of the osmotic droplet (C_o), then the coefficient P_f for bilayer water permeability may be derived from eqn. 2 from the slope of the curve obtained by plotting $(V/V_o)^2$ as a function of time. All data points presented in this paper are an average (n ≥ 50) of individual permeability runs, each of which took place over a time course (~5 min) for osmotic water movement across the droplet bilayer, during which time the droplet contact area (A) remains constant. The recorded videos and images were post-analyzed to measure the dimension of droplets and contact area using custom built image analysis software. All droplet pairs had substantially the same initial size relative to each other, in the diameter range of 100 ± 5 μm diameter.

DSC thermotropic data

Table S1. Temperature (T_m) and enthalpy (multilamellar liposome ΔH), for main phase transition of DOPC MLVs at varying concentrations of serotonin.

Total lipid to serotonin (mol)	DOPC MLVs		
	T_m (°C)	ΔH (kcal/mol)	FWHM (°C)
Control	–16.68 ± 0.28	8.34 ± 0.05	1.50 ± 0.01
100 to 1	–16.82 ± 0.02	8.23 ± 0.01	1.51 ± 0.01
50 to 1	–16.80 ± 0.11	8.17 ± 0.47	1.87 ± 0.01
30 to 1	–16.91 ± 0.29	7.78 ± 0.85	1.65 ± 0.01
10 to 1	–17.43 ± 0.32	6.46 ± 1.03	1.80 ± 0.01
4 to 1	–17.78 ± 0.65	5.31 ± 0.84	2.51 ± 0.02

Table S2. Temperature (T_m) and enthalpy (multilamellar liposome ΔH), for main phase transition of DOPC:DOPS (10:1) MLVs at varying concentrations of serotonin.

Total lipid to serotonin (mol)	DOPC:DOPS MLVs		
	T_m (°C)	ΔH (kcal/mol)	FWHM (°C)
Control	–15.88 ± 0.21	9.10 ± 0.67	0.95 ± 0.01
100 to 1	–16.33 ± 0.07	9.01 ± 0.21	1.41 ± 0.01
50 to 1	–16.30 ± 0.23	8.95 ± 0.72	1.46 ± 0.01
30 to 1	–16.67 ± 0.11	7.93 ± 0.87	1.81 ± 0.01
10 to 1	–17.34 ± 0.26	6.04 ± 0.90	2.39 ± 0.02
4 to 1	–18.98 ± 0.85	3.30 ± 0.65	3.74 ± 0.03

Table S3. Temperature (T_m) and enthalpy (multilamellar liposome ΔH), for main phase transition of DOPC:SM:Chol (1:1:0.2) MLVs at varying concentrations of serotonin.

Total lipid to serotonin (mol)	DOPC: SM: Chol MLVs		
	T_m (°C)	ΔH (kcal/mol)	FWHM (°C)
Control	–18.67 ± 0.33	2.67 ± 0.15	2.73 ± 0.01
100 to 1	–18.92 ± 0.31	2.46 ± 0.21	3.19 ± 0.02
50 to 1	–19.12 ± 0.34	2.33 ± 0.28	3.24 ± 0.02
30 to 1	–19.51 ± 0.44	2.05 ± 0.50	3.36 ± 0.02
10 to 1	–22.36 ± 1.33	1.17 ± 0.43	3.81 ± 0.03
4 to 1	–24.54 ± 1.90	0.45 ± 0.31	5.50 ± 0.31

Raman spectroscopic data

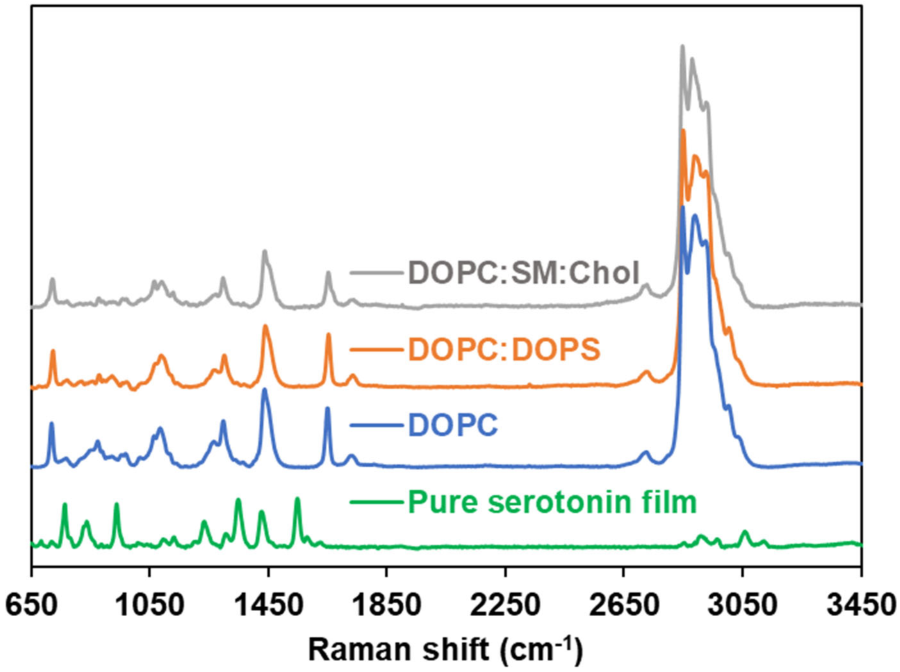

Figure S1. A representative Raman spectra (normalized at ~2848 cm^{-1}) of the pure DOPC, DOPC:DOPS (10:1), and DOPC:SM:Chol (1:1:0.2), along with pure serotonin film. Raman peak assignments for DOPC are shown in Table S4. The characteristic serotonin peak in the Raman spectrum appears at approximately 1540 cm^{-1}, corresponding to the indole ring stretching vibration.[3, 4]

Table S4. Peak assignments of DOPC Raman spectrum[5, 6]

Raman peak (cm^{-1})	Vibrational assignment
717	N-CH_3 symmetric stretch
872	N-CH_3 asymmetric stretch
1066	C–C trans stretch
1085	C–C gauche stretch
1299	CH_2 twist
1438	CH_2 bend
1655	C=C stretch
1735	C=O stretch
2849	CH_2 symmetric stretch
2891	CH_2 asymmetric stretch
2928	CH_3 symmetric stretch
3004	unsaturated C–H stretch
3034	Choline CH_3 asymmetric stretch

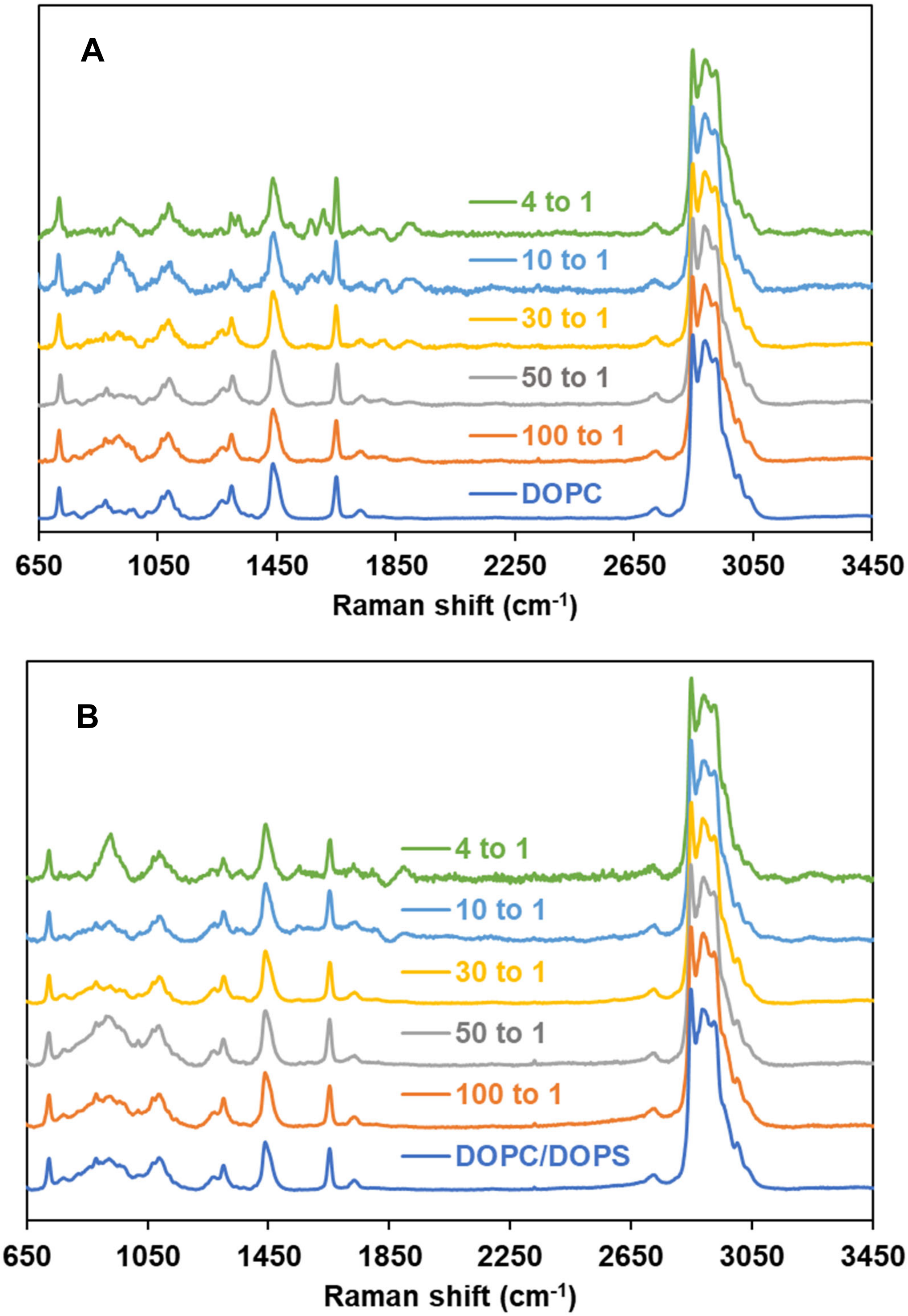
A
4 to 1
10 to 1
30 to 1
50 to 1
100 to 1
DOPC
650
1050
1450
1850
2250
2650
3050
3450
Raman shift (cm-1)
B
4 to 1
10 to 1
30 to 1
50 to 1
100 to 1
DOPC/DOPS
650
1050
1450
1850
2250
2650
3050
3450
Raman shift (cm-1)

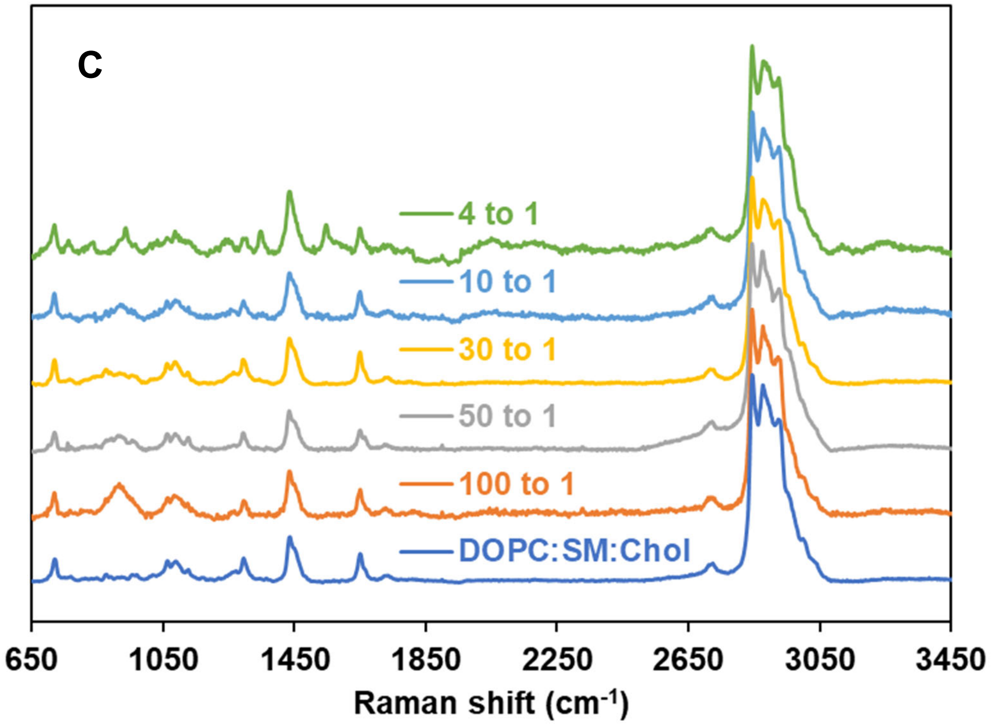

Figure S2. A representative Raman spectra (normalized at ~2848 cm^{-1}) of the (A) DOPC, (B) DOPC:DOPS (10:1), and (C) DOPC:SM:Chol (1:1:0.2) in the absence (blue) and presence of varying amount of serotonin (from 100 to 1 to 4 to 1 molar ratio of lipids to serotonin).

Table S5. The corresponding Raman intensity ratios (I) of [C–H_{term} (2930)/C–H_{asym} (2890)] of membranes of different compositions at room temperature as a function of serotonin content.

Lipids:serotonin (mol:mol)	Raman intensity ratio, I=2930/2890		
	DOPC	DOPC:DOPS (10:1)	DOPC:SM:Chol (1:1:0.2)
1:0	0.900 ± 0.012	0.883 ± 0.003	0.840 ± 0.010
100:1	0.901 ± 0.006	0.903 ± 0.008	0.860 ± 0.008
50:1	0.905 ± 0.014	0.928 ± 0.011	0.893 ± 0.006
30:1	0.916 ± 0.008	0.939 ± 0.013	0.904 ± 0.018
10:1	0.924 ± 0.019	0.953 ± 0.016	0.916 ± 0.015
4:1	0.927 ± 0.012	0.945 ± 0.002	0.900 ± 0.029

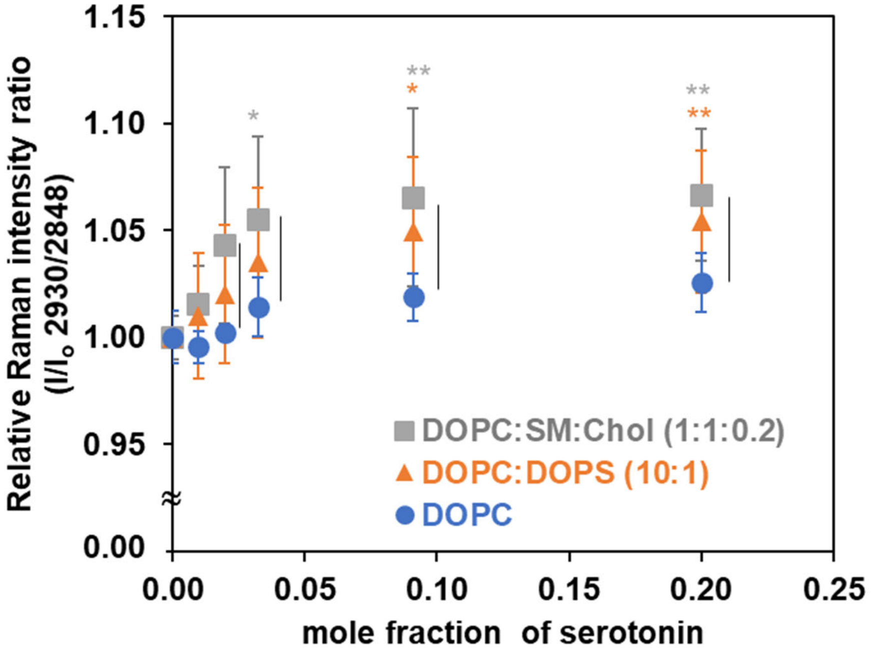

Figure S3. The relative Raman intensity ratios (I/I$_o$) of [C–H$_{term}$ (2930)/C–H$_{sym}$ (2850)] of membranes at room temperature as a function of serotonin content. Each data point represents average and standard deviation (SD) for three independently prepared samples. Three different regions are scanned for each sample and the average values are reported. All values are normalized to their respective control means. The data were analyzed by two-way ANOVA with Tukey's post hoc tests to compare the Raman intensity ratio across serotonin concentrations and their corresponding controls within each membrane model (colored stars) and among the three membrane models at the same serotonin concentration (black vertical lines). Only significant difference ($p < 0.05$) are indicated: colored stars, *$p < 0.05$ and **$p < 0.01$; black vertical lines for $p < 0.05$.

Table S6. The corresponding Raman intensity ratios (I) of [C–H$_{term}$ (2930)/C–H$_{sym}$ (2850)] of membranes of different compositions at room temperature as a function of serotonin content.

Lipids:serotonin (mol:mol)	Raman intensity ratio, I=2930/2850		
	DOPC	DOPC:DOPS (10:1)	DOPC:SM:Chol (1:1:0.2)
1:0	0.870 ± 0.012	0.840 ± 0.003	0.800 ± 0.010
100:1	0.866 ± 0.006	0.849 ± 0.001	0.812 ± 0.013
50:1	0.872 ± 0.003	0.857 ± 0.010	0.834 ± 0.029
30:1	0.883 ± 0.012	0.869 ± 0.015	0.844 ± 0.031
10:1	0.886 ± 0.010	0.882 ± 0.013	0.852 ± 0.033
4:1	0.892 ± 0.012	0.886 ± 0.011	0.853 ± 0.024

Contact Angle Measurement

For measuring the contact angle (θ), two apposing iso-osmotic droplets are brought into contact. The contact angle is determined from microscopic video images of the adhering droplets by analyzing their geometry as described in Equation 3, using the geometric parameters illustrated in Figure S3A.

$$2\theta = sin^{-1}\left(\frac{r}{R_1}\right) + sin^{-1}\left(\frac{r}{R_2}\right) \qquad (3)$$

Here, R_1 and R_2 are the radii the respective droplets, and r is the radius of the contact zone between them. Figure 3B shows images of contact angle of a DIB pair (DOPC in squalene) in the presence of serotonin HCl in 0.1M NaCl (droplet diameter is approximately 100 µm). Reported contact angle values represent the average of ten or more independent measurements.

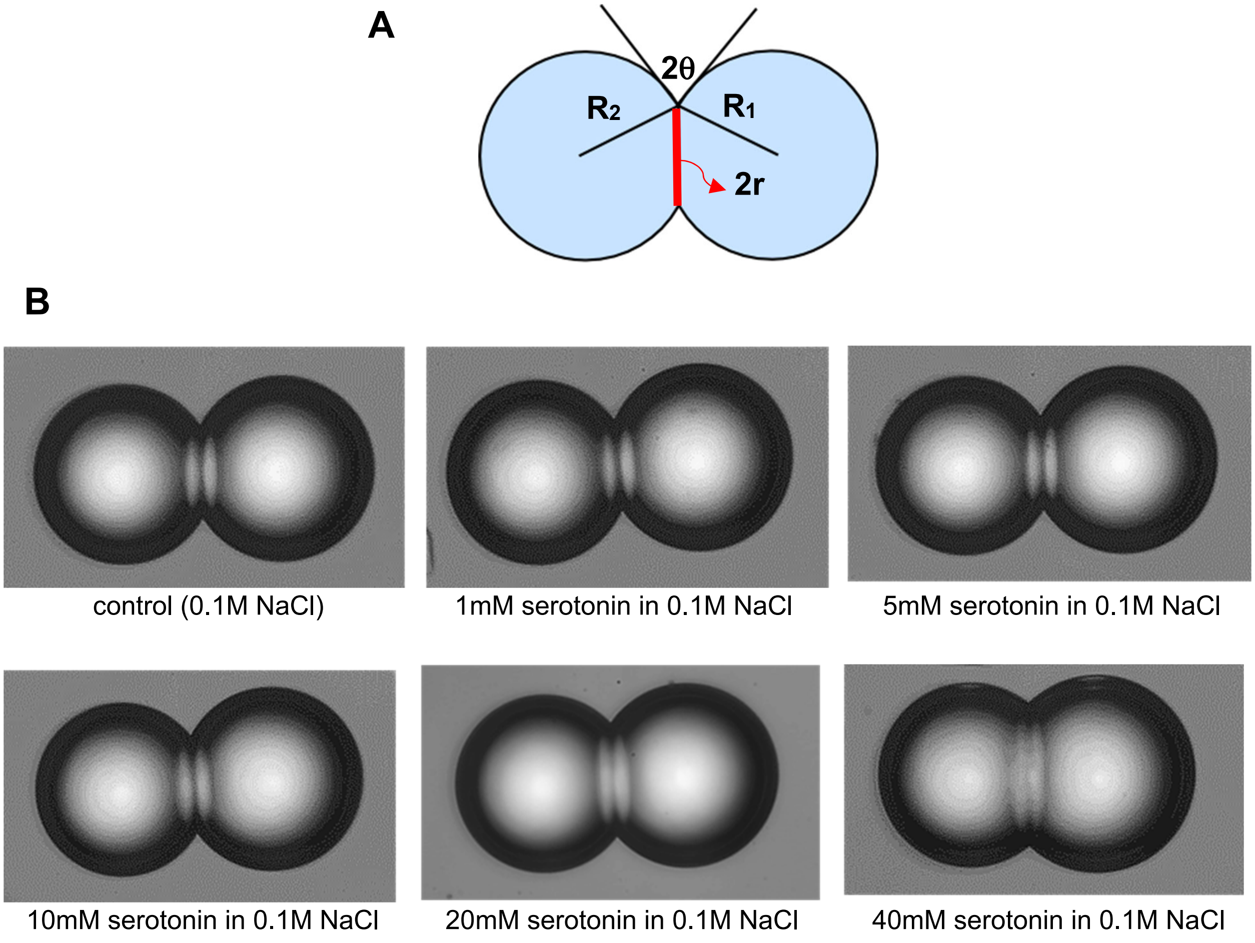

Figure S4. (A) A schematic of two adherent droplets illustrates the contact angle (θ), which is determined by equation 3, and (B) Images of contact angle of a DIB pair (DOPC in squalene) in the presence of serotonin HCl in 0.1M NaCl (droplet diameter is approximately 100 µm).

References

1. Lopez, M.; Evangelista, S. E.; Morales, M.; Lee, S. Enthalpic effects of chain length and unsaturation on water permeability across droplet bilayers of homologous monoglycerides. Langmuir 2017, 33 (4), 900-912.

2. Thiam, A. R.; Bremond, N.; Bibette, J. From stability to permeability of adhesive emulsion bilayers. Langmuir 2012, 28 (15), 6291-6298.

3. Manciu, F.S., Lee, K.H., Durrer, W.G. and Bennet, K.E., 2013. Detection and monitoring of neurotransmitters—A spectroscopic analysis. Neuromodulation: Technology at the Neural Interface, 16(3), pp.192-199

4. Manciu, F.S., Ciubuc, J.D., Sundin, E.M., Qiu, C. and Bennet, K.E., 2017. Analysis of Serotonin Molecules on Silver Nanocolloids—A Raman Computational and Experimental Study. Sensors, 17(7), p.1471.

5. Giancaspro, J., Scollan, P., Rosario, J., Miller, E., Braziel, S. and Lee, S., 2022. Structural determination of model phospholipid membranes by Raman spectroscopy: Laboratory experiment. Biochemistry and Molecular Biology Education, 50(2), pp.181-192.

6. Czamara, K., Majzner, K., Pacia, M.Z., Kochan, K., Kaczor, A. and Baranska, M., 2015. Raman spectroscopy of lipids: a review. Journal of Raman Spectroscopy, 46(1), pp.4-20.
